# Assisted reproductive technology induces different secondary sex ratio: parental and embryonic impacts

**DOI:** 10.1186/s12978-023-01723-8

**Published:** 2023-12-14

**Authors:** Jiansen Zhao, Haoran Shen, Qijiong Zhu, Jiong Liu, Jianhua Han, Ruiting Yi, Junxing Li, Yanshan Lin, Tao Liu, Xinqi Zhong

**Affiliations:** 1 Department of Neonatology, The Third Affiliated Hospital of Guangzhou Medical University, Guangdong Provincial Key Laboratory of Major Obstetric Diseases, Guangzhou, 510150 China; 2 China Greater Bay Area Research Center of Environment Health, School of Medicine, Jinan University, Guangzhou, 510006 China; 3 Department of Reproductive Center, The Third Affiliated Hospital of Guangzhou Medical University, Guangdong Provincial Key Laboratory of Major Obstetric Diseases, Guangzhou, 510150 China; 4 Department of Public Health and Preventive Medicine, School of Medicine, Jinan University, Guangzhou, 510632 China; 5 Nanshan School, Guangzhou Medical University, Guangzhou, 511436 China; 6 Department of Clinical Medicine, The Third Clinical School of Guangzhou Medical University, Guangzhou, 511436 China

**Keywords:** Assisted reproductive technology, Infertility, Logistic regression, Secondary sex ratio

## Abstract

**Background:**

Assisted reproduction technology (ART) has advanced significantly, raising concerns regarding its impact on the secondary sex ratio (SSR), which is the sex ratio at birth in offspring. This study aimed to explore factors affecting SSR in singletons, singletons from twin gestation, and twins from twin gestation within the context of ART.

**Methods:**

A retrospective analysis was conducted on data from 8335 births involving 6,223 couples undergoing ART. Binary logistic regression assessed relationships between parental and embryonic factors and SSR in singletons and singletons from twin gestation. Multinomial logistic regression models were utilized to identify factors influencing SSR in twins from twin gestation.

**Results:**

Secondary infertility (OR = 1.164, 95% CI: 1.009–1.342), advanced paternal age (OR = 1.261, 95% CI: 1.038–1.534), and blastocyst embryo transfer (OR = 1.339, 95% CI: 1.030–1.742) were associated with an increased SSR, while frozen embryo transfer (FET) showed a negative association with SSR (OR = 0.738, 95% CI: 0.597–0.912) in singletons. A longer duration of gonadotropin (Gn) usage reduced SSR in singletons (OR = 0.961, 95% CI: 0.932–0.990) and singletons from twin gestation (OR = 0.906, 95% CI: 0.838–0.980). In singletons from twin gestation, male-induced infertility (OR = 2.208, 95% CI: 1.120–4.348) and higher Gn dosage (OR = 1.250, 95% CI: 1.010–1.548) were significantly associated with an increased SSR. Women aged > 35 years and intracytoplasmic sperm injection (ICSI) were associated with lower SSR (OR = 0.539, 95% CI: 0.293–0.990 and OR = 0.331, 95% CI: 0.158–0.690, respectively). In twins from twin gestation, paternal age exceeded maternal age (OR = 0.682, 95% CI: 0.492–0.945) and higher Gn dosage (OR = 0.837, 95% CI: 0.715–0.980) were associated with a higher proportion of male twins. Cleavage stage transfer (OR = 1.754, 95% CI: 1.133–2.716) resulted in a higher percentage of boy-girl twins compared to blastocyst transfer.

**Conclusion:**

This study demonstrates the complex interplay of various factors in determining the SSR in ART, highlighting the importance of considering infertility type, paternal age, fertilization method, embryo transfer stage, and Gn use duration when assessing SSR. Nevertheless, further research with a large sample size is necessary to confirm and expand upon the findings of this study.

**Supplementary Information:**

The online version contains supplementary material available at 10.1186/s12978-023-01723-8.

## Background

Assisted reproductive technology (ART) has evolved from a marvel innovation to a conventional medical treatment for infertility. Since the first successful in vitro fertilization (IVF) treatment in 1978, it was estimated that at least 5 million infants worldwide had been born by ART [1], making IVF the principal treatment for infertile women [2]. Intracytoplasmic sperm injection (ICSI), introduced in 1992, allows the direct injection of a single spermatozoon into the oocyte and has become a valuable alternative for couples suffering infertility, especially those affected by poor sperm quality and unsuccessful IVF attempts [3].

The human sex ratio, critical for gender balance, consists of primary sex ratio (PSR) and secondary sex ratio (SSR). The PSR can deviate from 1:1 in theory, sometimes reaching as high as 170 males to 100 female [4]. However, SSR affected by spontaneous abortion, premature delivery [5], sex preference [6] and many other factors, is generally around 105 males per 100 female births [7]. ART procedures may influence SSR due to embryo handling. Researchers have tried to identify factors affecting SSR during ART [8, 9]. Dean J H et al. performed a population-based retrospective study indicating that ART procedures significantly influenced SSR [10]. They found that ICSI was related to a decrease in SSR compared to IVF, consistent with other studies [11, 12]. Frozen embryo transfer (FET) was also linked to a lower proportion of male babies in a study [13], although conflicting results were reported in others [14, 15]. Furthermore, Dean J H et al. [10] found that blastocyst stage transfer was associated with a higher proportion of male infants compared to cleavage stage transfer. A study by Maalouf, Walid E et al. [16] involving 85,511 treatment cycles and 106,066 babies in the United Kingdom, demonstrated that the neonatal SSR obtained from embryos transferred at the blastocyst stage is 6% higher than that of embryos transferred at the cleavage stage. Maternal age was considered an independent factor that influenced SSR, with women < 35 years more likely to have male births and women ≥ 35 years tending to have female infants [17]. However, Rueness, Janne et al. [18] found no evidence of a connection between maternal age and human sex ratio. Frattarelli, John L et al. [19] demonstrated that paternal age might have indirectly affect on SSR through blastocyst formation rates. Various factors, such as the type of infertility [20], specific infertility factor [11], and body mass index (BMI) [21], have been suggested as potential risk factors. Obviously, factors affecting SSR are multifaceted.

Even though numerous studies have explored potential factors of SSR in the ART population and have observed skewed gender distribution in the ART population compared to naturally conceived population, conclusive findings about the influence of ART on SSR remain elusive. Additionally, few studies have investigated SSR in the context of singletons and multiple births based on the premise that all mothers received double embryo transfer. Furthermore, there has been no investigation into the SSR of singletons born from twin pregnancies. Thus, this retrospective study, including 6223 cycles and 8335 babies, was conducted to investigate potential factors influencing the SSR of singletons, singletons from twin gestation, and twins from twin gestation. Singletons denote infants born from a single pregnancy where the mother carried only one child. Singletons from twin gestation refer to cases in which mothers with twin pregnancies successfully delivered only one child, while twins from twin gestation pertain to situations in which mothers with twin pregnancies successfully delivered two children.

## Methods

### Study subjects

**Fig. 1:**
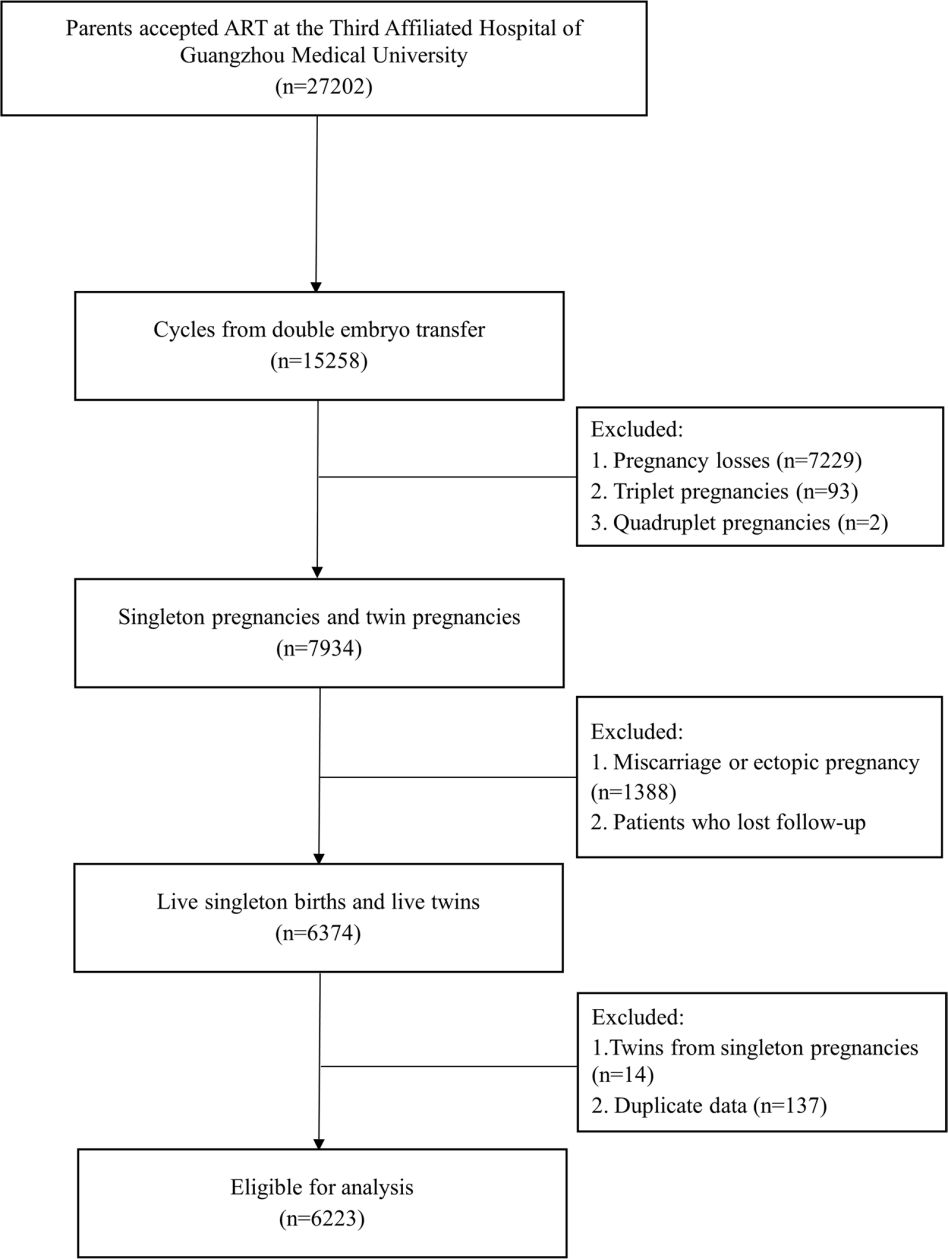
Flow diagram for the study selection. *ART* Assisted reproductive technology

A total of 27,202 couples who underwent assisted reproductive technology (ART) at the Centre for Reproductive Medicine of the Third Affiliated Hospital of Guangzhou Medical University, China, between January 2010 and January 2015, were recruited in the study. The inclusion criteria were as follows: (1) couples who received ART treatment at the Third Affiliated Hospital of Guangzhou Medical University; (2) ART cycles from double embryo transfer; (3) singleton pregnancies and twin pregnancies; (4) live births of singleton or twins; (5) subjects who completed the study follow-up. Moreover, for singleton pregnancies, only singleton births were included. The exclusion criteria are detailed in Fig. 1. Finally, a total of 6223 couples who underwent ART were selected as study subjects in the retrospective observational study.

### Data collection

In this research, various variables were examined, including the type of infertility, infertility factor, maternal age group, paternal age group, age disparities between partners, fertilization method, maternal body mass index (BMI), embryo transfer stage, dosage of gonadotropin (Gn), and days of Gn use. These variables were extracted from the hospital electronic medical records system of the Third Affiliated Hospital of Guangzhou Medical University. Besides, data regarding the gender of newborns was collected from the hospital’s electronic medical records system and telephonic follow-up.

The study received ethical approval from the ethics committee of the third Affiliated Hospital of Guangzhou Medical University. Informed consent was provided by all study participants.

### Definition and measurements

The secondary sex ratio (SSR) was defined as the ratio of male to female births in this study. Maternal age was classified into either < 35 or ≥ 35 years [17]. Paternal age was divided into groups based on interquartile ranges. The maternal BMI was divided into three groups in accordance with Chinese population standards: BMI < 18.5 kg/m^2^, 18.5 < BMI < 23.9 kg/m^2^, and BMI ≥ 24 kg/m^2^, according to the Chinese criteria[22]. Age differences referred to the difference between the mother's age and the father's age (maternal age minus paternal age). Embryos from in vitro fertilization (IVF) and intracytoplasmic sperm injection (ICSI) were all fresh at the time of transfer, while any remaining embryos were from frozen embryo transfer (FET). Infertility was categorized into primary and secondary infertility, according to whether a woman had previously experienced a pregnancy. Secondary infertile was characterized by women who had previously given a live birth and were now experiencing difficulty conceiving again [23].

### Data analysis

Shapiro–Wilk test was used to assess the normal distribution of continuous variables. For normally distributed continuous variables, t-tests were used, and the results were expressed as means with standard deviations. In cases where continuous variables did not meet a normal distribution, the Mann–Whitney U test was used, and continuous variables were presented as medians (25th percentile, 75th percentile). Categorical variables were presented as numbers and proportions. Based on the expectation value, the Pearson χ^2^ test or Fisher’s exact test would be used to compare differences between groups in categorical variables. Logistic regression models, adjusted for selected characteristics, were used to estimate the associations between parental and embryonic factors with SSR. Odds ratios (ORs) with a 95% confidence interval (CI) were calculated. All statistical analyses were conducted using SPSS software (version 25, IBM). A two-tailed P value < 0.05 was considered statistically significant.

## Results

### Baseline characteristics

In this study, a total of 8335 babies were included from 6223 cycles. The secondary sex ratio (SSR) of singletons, singletons from twin gestation, and twins from twin gestation were 124.1: 100, 134.2: 100 and 109.7: 100, respectively. For singletons, a significantly higher SSR was observed in births where parents had experienced secondary infertility compared to those with primary infertility (SSR: 136.5: 100 vs. 114.4: 100, P = 0.009). Additionally, paternal age ≥ 37 years was associated with a higher likelihood of live male births compared to other paternal age groups (SSR = 145.4: 100, P = 0.019). The median gonadotropin (Gn) usage was 2100 IU/L (25th-75th percentile: 1500–2775), and the median days of Gn use were 12 days (25th-75th percentile: 11–13). In singletons from twin gestation, the median Gn was 2025 IU/L (25th-75th percentile: 1462.4–2850), and the median days of Gn use were 12 days (25th-75th percentile: 11–13). For twins from twin gestation, there was a significantly higher proportion of males with blastocyst stage embryo transfer compared to cleavage stage transfers (SSR:138.6: 100 vs. 103.3: 100, P = 0.002). In addition, the median Gn usage for this group was 2062.5 IU/L (25th-75th percentile: 1575–2700), with a median duration of Gn use of 12 days (25th-75th percentile: 11–13) (Table 1).

**Table 1:** Basic characteristics of the patients and embryos undergoing ART treatment

Variables	Singletons			Singletons from twin gestation			Twins from twin gestation		
	Male	Female	P	Male	Female	P	Male	Female	P
Infertility type (n, %)			0.009			0.524			0.959
Primary	1018(53.4)	890(46.6)		163(56.0)	128(44.0)		1182(52.1)	1086(47.9)	
Secondary	947(57.7)	694(42.3)		159(58.7)	112(41.3)		1028(52.6)	928(47.4)	
Infertility factor (n, %)			0.463			0.791			0.278
Male	1324(56.0)	1042(44.0)		225(57.7)	165(42.3)		1564(53.0)	1388(47.0)	
Female	314(55.3)	254(44.7)		46(59.0)	32(41.0)		296(48.7)	312(51.3)	
Both male and female	327(53.2)	288(46.8)		51(54.3)	43(45.7)		350(52.7)	314(47.3)	
Maternal age group (n, %)			0.085			0.230			0.823
≤35	1748(54.9)	1437(45.1)		286(58.2)	205(41.8)		2102(52.3)	1914(47.7)	
>35	217(59.6)	147(40.4)		36(50.7)	35(49.3)		108(51.9)	100(48.1)	
Paternal age group (n, %)			0.019			0.968			0.864
≤30	519(52.5)	470(47.5)		85(55.9)	67(44.1)		717(52.0)	663(48.0)	
31–33^1^	545(56.6)	418(43.4)		83(58.5)	59(41.5)		418(51.2)	398(48.8)	
34–36^2^	424(53.5)	368(46.5)		86(58.1)	62(41.9)		547(53.4)	477(46.6)	
≥37^3^	477(59.3)	328(40.7)		68(56.7)	52(43.3)		528(52.7)	476(47.3)	
Age difference (n, %)			0.675			0.330			0.093
Older mother	302(53.7)	260(46.3)		46(55.4)	37(44.6)		304(49.4)	312(50.6)	
Older father	1400(55.8)	1110(44.2)		239(59.0)	166(41.0)		1601(53.2)	1411(46.8)	
None	263(55.1)	214(44.9)		37(50.0)	37(50.0)		305(51.2)	291(48.8)	
Fertilization method (n, %)			0.526			0.186			0.238
IVF	1178(56.2)	919(43.8)		201(59.3)	138(40.7)		1394(52.1)	1280(47.9)	
ICSI	298(55.1)	243(44.9)		29(46.8)	33(53.2)		252(48.5)	268(51.5)	
FET	455(53.9)	389(46.1)		89(57.8)	65(42.2)		522(54.7)	432(45.3)	
Maternal BMI (n, %)			0.284			0.805			0.359
<18.5	275(52.5)	249(47.5)		37(54.4)	31(45.6)		308(50.5)	302(49.5)	
18.5–23.9	1351(56.2)	1054(43.8)		228(58.2)	164(41.8)		1530(52.4)	1390(47.6)	
≥24	339(54.7)	281(45.3)		57(55.9)	45(44.1)		372(53.6)	322(46.4)	
Type of cycle (n, %)			0.332			0.884			0.240
Fresh	1510(55.8)	1195(44.2)		233(57.1)	175(42.9)		1688(51.6)	1582(48.4)	
Frozen	454(53.9)	388(46.1)		89(57.8)	65(42.2)		522(54.7)	432(45.3)	
Embryo transfer stage (n, %)			0.378			0.638			0.002
Cleavage stage	1694(55.0)	1387(45.0)		255(58.1)	184(41.9)		1741(50.8)	1685(49.2)	
Blastocyst	227(57.3)	169(42.7)		64(55.7)	51(44.3)		424(58.1)	306(41.9)	
Usage of Gn (IU/L, M (P25, P75))	2100(1500, 2775)			2025(1462.4, 2850)			2062.5(1575, 2700)		
Days of Gn use (day, M (P25, P75))	12(11, 13)			12(11, 13)			12(11, 13)		

*ART* Assisted reproductive technology, *ICSI* Intracytoplasmic sperm injection, *IVF* In vitro fertilization, *FET* Frozen embryo transfer, *Gn* Gonadotropin, *BMI* Body mass index

^1^Singletons from twin gestation and twins from twin gestation were classified as 31–33 and 31–32, respectively

^2^Singletons from twin gestation and twins from twin gestation were classified as 34–37 and 33–35, respectively

^3^Singletons from twin gestation and twins from twin gestation were classified as ≥ 38 and ≥ 36, respectively

### Factors associated with SSR in singletons

For singletons, univariable logistic regression analyses revealed associations between SSR and factors such as infertility type, paternal age, and days of Gn (Additional file 1). Following mutual adjustment for infertility type, infertility factors, paternal age, fertilization methods, embryo transfer stages and days of Gn use, higher SSR was associated with patients experiencing secondary infertility (OR = 1.164, 95% CI:1.009–1.342) and fathers aged 37 years or older (OR = 1.261, 95% CI: 1.038–1.534); Blastocyst embryo transfer exhibited a higher SSR compared to cleavage stage embryo transfer (OR = 1.339, 95% CI: 1.030–1.742). Lower SSR (OR = 0.738, 95% CI: 0.597–0.912) was observed in patients undergoing frozen embryo transfer (FET) treatment compared to vitro fertilization. A slight decline in SSR was noted with an increase in the duration of Gn use (in days) (OR = 0.961, 95% CI: 0.932–0.990) (Table 2).

**Table 2:** Multivariable logistic regression analysis with SSR in singletons

Variables	OR	95% CI
Infertility type		
Primary	1.000	–
Secondary	1.164	1.009–1.342
Infertility factor		
Female	1.000	–
Male	1.031	0.800–1.330
Both male and female	0.896	0.739–1.087
Paternal age group (year)		
≤30	1.000	–
31–33	1.143	0.951–1.372
34–36	1.048	0.865–1.271
≥37	1.261	1.038–1.534
Fertilization method		
IVF	1.000	–
ICSI	0.996	0.772–1.285
FET	0.738	0.597–0.912
Embryo transfer stage		
Cleavage stage	1.000	–
Blastocyst	1.339	1.030–1.742
Days of Gn use (day)	0.961	0.932–0.990

Male singletons selected as the reference category

*SSR* Secondary sex ratio, *IVF* In vitro fertilization, *ICSI* Intracytoplasmic sperm injection, *FET* Frozen embryo transfer, *Gn* Gonadotropin

### Factors associated with SSR in singletons from twin gestation

For singletons from twin gestation, initial univariable logistic regression analyses did not reveal any significant difference between SSR and potential factors in (Additional file 2). However, subsequent multivariable regression analysis, after adjusting for confounding factors, showed that infertility attributed to male factors was positively associated with an increased SSR (OR = 2.208, 95% CI: 1.120–4.348) in the model. Moreover, SSR was significantly different between the reference category (age ≤ 35 years) and women aged > 35 years (OR = 0.539, 95% CI: 0.293–0.990). Besides, intracytoplasmic sperm injection (ICSI) accounted for a decreased SSR (OR = 0.331, 95% CI: 0.158–0.690) compared to in vitro fertilization (IVF). Increased duration of Gn use also significantly linked to a decreased the SSR (OR = 0.906, 95% CI: 0.838–0.980). Moreover, a higher dosage of Gn was related to an increased SSR (OR = 1.250, 95% CI: 1.010–1.548) (Table 3).

**Table 3:** Multivariable logistic regression analysis from twin gestation

Variables	OR	95% CI
Infertility factor		
Female	1.000	
Male	2.208	1.120–4.348
Both male and female	0.989	0.612–1.597
Maternal age group (year)		
≤35	1.000	
>35	0.539	0.293–0.990
Paternal age group (year)		
≤30	1.000	
31–33	1.135	0.706–1.821
34–37	1.235	0.764–1.996
≥38	1.330	0.762–2.326
Fertilization method		
IVF	1.000	
ICSI	0.331	0.158–0.690
FET	0.938	0.595–1.479
Dosage of Gn (× 10^3^ IU/L)	1.250	1.010–1.548
Days of Gn use (day)	0.906	0.838–0.980

Male births selected as the reference category

*IVF* In vitro fertilization, *ICSI* Intracytoplasmic sperm injection, *FET* Frozen embryo transfer, *Gn* Gonadotropin

### Factors associated with SSR in twins from twin gestation

In the case of twins born from twin gestation, only age difference and embryo transfer stage were found to be associated with SSR (Additional file 3). The results of the multinomial logistic regression modeling of the combined effect of dosage of Gn, days of Gn use, age difference, paternal age group, maternal body mass index (BMI), and embryo transfer stage of SSR among twins from twin gestation, are shown in Table 4. In comparison to groups with no age difference, groups in which paternal age exceeded maternal age exhibited a higher proportion of male twins relative to boy-girl twins (OR = 0.682, 95% CI: 0.492–0.945). Cleavage stage transfer had a higher probability of having boy-girl twins compared to blastocyst transfer (OR = 1.754, 95% CI: 1.133–2.716). In addition, a higher dosage of Gn was associated with a greater percentage of male twins (OR = 0.837, 95% CI: 0.715–0.980).

**Table 4:** Multinomial logistic regression analysis with SSR in twins from twin gestation

Birth^*^	Variables	OR	95% CI
Female twins	Dosage of Gn (× 10^3^ IU/L)	0.931	0.805–1.076
	Days of Gn use (day)	1.021	0.977–1.067
	Age difference		
	Older mother	0.815	0.546–1.215
	Older father	0.864	0.629–1.187
	None	1.000	
	Embryo transfer stage		
	Cleavage stage	0.783	0.530–1.158
	Blastocyst	1.000	
	Paternal age group (year)		
	≤30	1.056	0.773–1.444
	31–32	0.988	0.712–1.371
	33–35	1.072	0.794–1.448
	≥36	1.000	
	Maternal BMI		
	<18.5	1.129	0.770–1.654
	18.5–23.9	1.048	0.786–1.396
	≥24	1.000	
	Fertilization method		
	IVF	1.061	0.703–1.600
	ICSI	0.844	0.514–1.386
	FET	1.000	
Boy-girl twins	Dosage of Gn (× 10^3^ IU/L)	0.837	0.715–0.980
	Days of Gn use (day)	1.032	0.985–1.081
	Age difference		
	Older mother	0.917	0.616–1.366
	Older father	0.682	0.492–0.945
	None	1.000	
	Embryo transfer stage		
	Cleavage stage	1.754	1.133–2.716
	Blastocyst	1.000	
	Paternal age group (year)		
	≤30	0.939	0.672–1.312
	31–32	0.972	0.686–1.377
	33–35	0.995	0.719–1.377
	≥36	1.000	
	Maternal BMI		
	<18.5	1.340	0.892–2.012
	18.5–23.9	1.160	0.845–1.591
	≥24	1.000	
	Fertilization method		
	IVF	0.846	0.546–1.312
	ICSI	0.882	0.531–1.464
	FET	1.000	

^*^Male twins selected as the reference category

*SSR* Secondary sex ratio, *IVF* In vitro fertilization, *ICSI* Intracytoplasmic sperm injection, *FET* Frozen embryo transfer, *Gn* Gonadotropin, *BMI* Body mass index

## Discussion

The secondary sex ratio (SSR) is an important implication for population health and fertility. In this hospital-based retrospective study, we investigated the factors related to the sex ratio of newborns born to mothers who underwent assisted reproductive technology (ART) and achieved singleton or twin deliveries. Our findings shed light on various factors that influence SSR in singletons and twins, underscoring the impact of a specific ART protocol on sex ration of newborns. These factors included infertility type, paternal age group, fertilization method, embryo transfer stage, course of gonadotropin (Gn), infertility factor, maternal age group, and age difference.

In terms of the infertility type, our findings suggest that, in singleton pregnancies, secondary infertility is more prone to having male offspring through ART compared to primary infertility. Primary infertility typically affects young patients who have never experienced a pregnancy. The causes of infertility in this group are often complex and diverse, making them more susceptible to sperm-ovum fertilization failure and low fertilization rates during their first in vitro fertilization (IVF)-assisted pregnancy [20]. Our research also indicates an association between SSR and paternal age. Studies have shown that, in a singleton pregnancy, boys are more likely to be born to older fathers than in the control group [15]. As society progresses and reproductive technology advances, the number of older couples seeking ART is on the rise. Our research indicates that fathers aged 37 or above have a higher probability of having male babies compared to fathers aged 30 or less. However, Jacobsen R [24] found that a lower SSR was associated with an increase in paternal age, possible due to a significant reduction in blastocyst formation rate over paternal age [25]. This reduction may lead to a higher proportion of male embryos in blastocysts [26], ultimately affecting the sex ratio at birth among older men. Thus, the relationship between SSR and paternal age warrants further study. In addition, in singletons from twin gestation, maternal age was also significantly associated with SSR. Those maternal ages > 35 were related to a reduced SSR compared to those aged 35 or younger. Tarín J J et al. [17] suggested a significant shift toward females (71.4%) in the sex ratio in mothers aged 35 or older, while the neonatal sex ratio was significantly shifted toward males (62.7%) in women under 35. The mechanism behind this may be linked with higher levels of stress in older populations, with female embryos exhibiting greater stress tolerance, leading to the elimination of more male embryos [18].

In addition, the characteristics of the ART treatment can alter the proportion of male infants in our research. Previous studies show that the use of BT is associated with an increase in SSR, while intracytoplasmic sperm injection (ICSI) is related to a reduction in SSR [27]. However, only a few studies have reported the difference between SSR in fresh embryo transfer and frozen embryo transfer (FET). In particular, our research shows that the use of FET significantly increases SSR compared to the use of IVF. Several biologically plausible mechanisms may explain the selective survival of male embryos in multiple embryo transfer, including immunologic factors, genetic survival advantages encoded on the Y chromosome, and imprinting errors on the X chromosomes of female embryos [13]. Most Chinese reproductive medicine centers use blastocyst transfers in FET cycles. It is suspected that the alteration of SSR toward males is due to the blastocyst transfer itself rather than FET [14].

Our study revealed that prolonged gonadotropin use was linked to a higher proportion of female infants in both singletons and singletons from twin gestation. The underlying reasons for the discrepancy remain unclear. However, it is unlikely that prolonged gonadotropin use would cause sex-selective abortions by adversely affecting male-fertilized eggs [28]. Thus, further studies are warranted to explore the potential association between SSR and Gn.

Consistent with existing literature, blastocyst embryos were found to be correlated with a higher proportion of male offspring in contrast with cleavage stage embryos. Male embryos tend to develop faster than female embryos do in IVF or ICSI, and as a result, operators are more inclined to select embryos with fast embryonic development and favorable morphology for transfer [29, 30]. Moreover, the male embryo may be likely to achieve the blastocyst embryo transfer [31]. Another explanation for the elevated SSR following blastocyst stage transplantation could be the atypical inactivation of one of the two X chromosomes in female embryos [32]. Preferential female mortality at early post-implantation stages may be due to in-vitro-culture-induced precocious X-chromosome inactivation, combined with a decrease induced by ICSI in the number of trophectoderm cells in female blastocysts [33]. In addition, the difference in sex ratio may also be attributed to the fact that cleavage-stage embryo transfer results in a lower percentage of biochemical pregnancy losses per embryo transfer compared to blastocyst transfer [27].

For singletons from twin gestation, except for the same impact of gonadotropin days on the sex ratio of the newborn, there are additional factors on the sex ratio of the newborn. Our research aligns with several studies [10, 34] that have highlighted the connection between using ICSI and a reduction in SSR. ICSI is typically performed to address male factor infertility, often related to poor semen quality or morphological defects of semen [12] and spermatozoa that carried the Y-chromosome suffered from morphological alterations as well as the reduction of the number [35]. As a result, the use of ICSI could lead to selection bias because operators were more likely to select sperm that exhibit normal morphology and healthy [35, 36]. Additionally, relevant researches have demonstrated that oocytes are more likely to accept Y-bearing spermatozoa for fertilization in IVF as opposed to ICSI [33, 37]. This could account for the observed lower SSR in the ICSI treatment group compared to IVF.

Regarding infertility factors, our study identified a higher percentage of male babies born to ART patients solely attributed to male factors compared to those with other infertility factors. However, Dean J H et al. [10] claimed no association between infertility factors and SSR. Likewise, Luke, Barbara et al. [11] found that male factor subfertility did not influence SSR after adjusting for potential confounders. Thus, further studies are still needed to explore the relationship between infertility factors and SSR.

For twins from twin gestation, our results suggest that older fathers tend to have a higher chance of having twin boys. Nevertheless, further data is needed to support our findings. Several factors may be associated with SSR in this context. Firstly, older fathers are more likely to have boys. A cross-sectional study conducted in China supported this observation, revealing that the SSR in the oldest group of fathers was higher than that of the youngest age group, which was consistent with our results [34]. However, a retrospective study in China shows that, consistent with spontaneous pregnancy, paternal age over 32 has an imbalance towards lower SSR [27]. Previous study has shown a decrease in the blastocyst formation rate with an increase in parental age [19]. Secondly, younger mothers are more likely to give birth to boys. Rueness et al. pointed out in their study that there was no statistical difference between maternal age and SSR in the general pregnant population. However, in pregnant women diagnosed with preeclampsia, as the age of pregnant women increases, the proportion of male infants born decreases. This suggests that the sex ratio shifts when older pregnant women are affected by complex pregnancy conditions [18].

Furthermore, this study is the first time to suggest that cleavage stage transfer may have a positive impact on the birth of boy-girl twins, an interesting finding as this phenomenon has not been previously reported in the literature. However, this finding might be due to chance, given the relatively small sample size of the study. A larger sample size and more rigorous multi-center collaboration are needed to further investigate the reasons for this result.

At present, many parents undergoing ART treatments hold preferences for gender of their offspring. However, Chinese law prohibits gender selection through ART. Nonetheless, clinicians can provide patients with an expected probability based on this study. Moreover, it is essential to recognize that the global fertility rate is continuously decreasing, and an increasing number of infertile individuals are opting for ART treatment. Consequently, the impact of the SSR on population gender balance will become increasingly pronounced. Clinicians can play a key role in mitigating any gender imbalances by considering to the potential influencing factors discussed in this study.

## Conclusions

Three models were constructed to explore the potential association between SSR and various factors related to both parents and embryos. Several factors were found that had a significant effect on SSR. In singletons, secondary infertility, paternal age ≥ 37 years and blastocyst transfer were linked with a higher likelihood of male births compared to primary infertility, paternal age < 37 years, and cleavage stage transfer, respectively, Conversely, FET and increased duration of Gn use were linked to a decreased SSR. For singletons from twin gestation, male-induced infertility, and a higher dosage of Gn were positively correlated with SSR, while maternal age > 35 years, ICSI, and the duration of Gn use were associated with a decrease of SSR compared to maternal age ≤ 35 years and IVF, respectively. In twins from twin gestation, groups where paternal age exceeded maternal age were more likely to have male twins than to have boy-girl twins compared to groups with no age difference. Cleavage stage transfer had a higher probability of resulting in boy-girl twins compared to blastocyst transfer. While the study acknowledges the need for more extensive sample size for future research, the impacts of infertility factors, age difference and duration of Gn use on SSR remain important considerations. Understanding which factors within ART process can affect SSR holds significance for maintaining gender balance in the population.

## Limitations

This study has several limitations. Firstly, our samples were derived from a single reproductive center, and our sample size is relatively small. Thus, to validate present findings, it’s necessary to expand the sample size in future analyses. Secondly, due to the retrospective nature of this analysis, there are inherent limitations concerning the integrity and homogeneity of the data. Lastly, the data was extracted based on the hospital's system, without the use of a detailed questionnaire survey. This method may lead to the missing of certain important information. In the future, we plan to perform multicenter research to satisfy sample diversity and meet sample capacity requirements, enhancing the richness and credibility of the research evidence. Besides, we intend to conduct laboratory research to figure out whether infertility factors, age difference and duration of Gn use indeed impact SSR and to explore the relevant mechanisms. With the improvement of our research, we will perform a more comprehensive analysis of the factors influencing SSR.

### Supplementary Information

**Additional file 1: Table S1.** Univariate logistic regression analyses of different variables with SSR in Singletons.

**Additional file 2: Table S2.** Univariate logistic regression analyses of different variables with SSR in Singletons from twin gestation.

**Additional file 3: Table S3.** Univariate logistic regression analyses of different variables with SSR in twins from twin gestation.

## Data Availability

The data will be made available on request to the corresponding author.

## Abbreviations

SSR: Secondary sex ratio
PSR: Primary sex ratio
ART: Assisted reproductive technology
ICSI: Intracytoplasmic sperm injection
IVF: In vitro fertilization
FET: Frozen embryo transfer
Gn: Gonadotropin
