# Supplementary material for: Assisted reproductive technology induces different secondary sex ratio: parental and embryonic impacts
Source: Reprod Health. 2023 Dec 14;20:184. doi: 10.1186/s12978-023-01723-8 (PMC10722851; doi:10.1186/s12978-023-01723-8)
Supplement: Supplementary file 1 — Additional file 1: Table S1. Univariate logistic regression analyses of different variables with SSR in Singletons. [file 12978_2023_1723_MOESM1_ESM.docx]

| **Additional file 1: Table S1 Univariate logistic regression analyses of different variables with SSR in Singletons.** | | |
| --- | --- | --- |
| **Variables** | **OR** | **95%CI** |
| **Infertility type** |  |  |
| Primary | 1.000 |  |
| Secondary | 1.193 | 1.045-1.362 |
| **Infertility factor** |  |  |
| Female | 1.000 |  |
| Male | 0.973 | 0.809-1.170 |
| Both male and female | 0.894 | 0.748-1.067 |
| **Maternal age group** |  |  |
| ≤35 | 1.000 |  |
| >35 | 1.214 | 0.973-1.513 |
| **Paternal age group** |  |  |
| ≤30 | 1.000 |  |
| 31-33 | 1.181 | 0.988-1.410 |
| 34-36 | 1.044 | 0.865-1.258 |
| ≥37 | 1.318 | 1.092-1.590 |
| **Age difference** |  |  |
| Older mother | 1.000 |  |
| Older father | 1.086 | 0.903-1.305 |
| None | 1.058 | 0.828-1.351 |
| **Fertilization method** |  |  |
| IVF | 1.000 |  |
| ICSI | 0.957 | 0.791-1.157 |
| FET | 0.912 | 0.777-1.072 |
| **Maternal BMI** |  |  |
| <18.5 | 1.000 |  |
| 18.5-23.9 | 1.160 | 0.961-1.403 |
| ≥24 | 1.093 | 0.865-1.379 |
| **Embryo transfer stage** |  |  |
| Cleavage stage | 1.000 |  |
| Blastocyst | 1.100 | 0.890-1.359 |
| **Dosage of Gn** | 1.027 | 0.973-1.083 |
| **Course of Gn** | 0.967 | 0.941-0.995 |
| SSR: Secondary sex ratio, IVF: In vitro fertilization, ICSI: Intracytoplasmic sperm injection, FET: Frozen embryo transfer, BMI: Body mass index, Gn: Gonadotropin | | |
