# Supplementary material for: Assisted reproductive technology induces different secondary sex ratio: parental and embryonic impacts
Source: Reprod Health. 2023 Dec 14;20:184. doi: 10.1186/s12978-023-01723-8 (PMC10722851; doi:10.1186/s12978-023-01723-8)
Supplement: Supplementary file 2 — Additional file 2: Table S2. Univariate logistic regression analyses of different variables with SSR in Singletons from twin gestation. [file 12978_2023_1723_MOESM2_ESM.docx]

| **Additional file 2: Table S2 Univariate logistic regression analyses of different variables with SSR in Singletons from twin gestation.** | | |
| --- | --- | --- |
| Variables | OR | 95%CI |
| **Infertility type** |  |  |
| Primary | 1.000 |  |
| Secondary | 1.115 | 0.797-1.558 |
| **Infertility factor** |  |  |
| Female | 1.000 |  |
| Male | 1.054 | 0.644-1.727 |
| Both male and female | 0.870 | 0.553-1.368 |
| **Maternal age group** |  |  |
| ≤35 | 1.000 |  |
| >35 | 0.737 | 0.448-1.214 |
| **Paternal age group** |  |  |
| ≤30 | 1.000 |  |
| 31-33 | 1.109 | 0.698-1.761 |
| 34-37 | 1.093 | 0.692-1.727 |
| ≥38 | 1.031 | 0.636-1.669 |
| **Age difference** |  |  |
| Older mother | 1.000 |  |
| Older father | 1.157 | 0.719-1.866 |
| None | 0.805 | 0.429-1.508 |
| **Fertilization method** |  |  |
| IVF | 1.000 |  |
| ICSI | 0.604 | 0.350-1.040 |
| FET | 0.940 | 0.639-1.383 |
| **Maternal BMI** |  |  |
| <18.5 | 1.000 |  |
| 18.5-23.9 | 1.164 | 0.694-1.953 |
| ≥24 | 1.062 | 0.573-1.969 |
| **Embryo transfer stage** |  |  |
| Cleavage stage | 1.000 |  |
| Blastocyst | 0.906 | 0.598-1.370 |
| **Dosage of Gn** | 1.037 | 0.900-1.195 |
| **Course of Gn** | 0.960 | 0.906-1.016 |
| SSR: Secondary sex ratio, IVF: In vitro fertilization, ICSI: Intracytoplasmic sperm injection, FET: Frozen embryo transfer, BMI: Body mass index, Gn: Gonadotropin | | |
