# Supplementary material for: Assisted reproductive technology induces different secondary sex ratio: parental and embryonic impacts
Source: Reprod Health. 2023 Dec 14;20:184. doi: 10.1186/s12978-023-01723-8 (PMC10722851; doi:10.1186/s12978-023-01723-8)
Supplement: Supplementary file 3 — Additional file 3: Table S3. Univariate logistic regression analyses of different variables with SSR in twins from twin gestation. [file 12978_2023_1723_MOESM3_ESM.docx]

| **Birth** | **Variables** | **OR** | **95%CI** |
| --- | --- | --- | --- |
| **Female twins** | **Infertility type** |  |  |
|  | Primary | 0.979 | 0.799-1.199 |
|  | Secondary | 1.000 |  |
|  | **Infertility factor** |  |  |
|  | Male | 0.912 | 0.689-1.207 |
|  | Female | 0.705 | 0.483-1.030 |
|  | Both male and female | 1.000 |  |
|  | **Maternal age group** |  |  |
|  | ≤35 | 1.145 | 0.713-1.838 |
|  | >35 | 1.000 |  |
|  | **Paternal age group** |  |  |
|  | ≤30 | 1.123 | 0.854-1.475 |
|  | 31-32 | 1.026 | 0.750-1.402 |
|  | 33-35 | 1.110 | 0.830-1.484 |
|  | ≥36 | 1.000 |  |
|  | **Age difference** |  |  |
|  | Older mother | 0.815 | 0.554-1.198 |
|  | Older father | 0.839 | 0.624-1.129 |
|  | None | 1.000 |  |
|  | **Fertilization method** |  |  |
|  | IVF | 0.886 | 0.695-1.130 |
|  | ICSI | 0.716 | 0.495-1.036 |
|  | FET | 1.000 |  |
|  | **Maternal BMI** |  |  |
|  | <18.5 | 1.149 | 0.796-1.659 |
|  | 18.5-23.9 | 1.101 | 0.835-1.451 |
|  | ≥24 | 1.000 |  |
|  | **Embryo transfer stage** |  |  |
|  | Cleavage stage | 0.809 | 0.625-1.045 |
|  | Blastocyst | 1.000 |  |
|  | **Dosage of Gn** | 0.946 | 0.863-1.036 |
|  | **Days of Gn use** | 0.994 | 0.966-1.022 |
| **Boy-girl twins** | **Infertility type** |  |  |
|  | Primary | 1.013 | 0.816-1.256 |
|  | Secondary | 1.000 |  |
|  | **Infertility factor** |  |  |
|  | Male | 0.883 | 0.654-1.191 |
|  | Female | 0.962 | 0.656-1.412 |
|  | Both male and female | 1.000 |  |
|  | **Maternal age group** |  |  |
|  | ≤35 | 1.119 | 0.678-1.845 |
|  | >35 | 1.000 |  |
|  | **Paternal age group** |  |  |
|  | ≤30 | 1.196 | 0.894-1.599 |
|  | 31-32 | 1.152 | 0.829-1.600 |
|  | 33-35 | 1.048 | 0.765-1.436 |
|  | ≥36 | 1.000 |  |
|  | **Age difference** |  |  |
|  | Older mother | 0.931 | 0.632-1.371 |
|  | Older father | 0.707 | 0.520-0.961 |
|  | None | 1.000 |  |
|  | **Fertilization method** |  |  |
|  | IVF | 1.083 | 0.827-1.419 |
|  | ICSI | 1.173 | 0.807-1.706 |
|  | FET | 1.000 |  |
|  | **Maternal BMI** |  |  |
|  | <18.5 | 1.504 | 1.020-2.218 |
|  | 18.5-23.9 | 1.238 | 0.913-1.679 |
|  | ≥24 | 1.000 |  |
|  | **Embryo transfer stage** |  |  |
|  | Cleavage stage | 1.480 | 1.084-2.020 |
|  | Blastocyst | 1.000 |  |
|  | **Dosage of Gn** | 0.912 | 0.828-1.006 |
|  | **Days of Gn use** | 0.993 | 0.964-1.023 |
| SSR: Secondary sex ratio, IVF: In vitro fertilization, ICSI: Intracytoplasmic sperm injection, FET: Frozen embryo transfer, BMI: Body mass index, Gn: Gonadotropin | | | |

**Additional file 3: Table S3 Univariate logistic regression analyses of different variables with SSR in twins from twin gestation.**
